# Why Don't Men Understand Women? Altered Neural Networks for Reading the Language of Male and Female Eyes

**DOI:** 10.1371/journal.pone.0060278

**Published:** 2013-04-10

**Authors:** Boris Schiffer, Christina Pawliczek, Bernhard W. Müller, Elke R. Gizewski, Henrik Walter

**Affiliations:** 1 Division of Forensic Psychiatry, Department of Psychiatry, Psychotherapy and Preventive Medicine, LWL-University Hospital Bochum, Germany; 2 Institute of Forensic Psychiatry, University of Duisburg-Essen, Germany; 3 Department of Psychiatry and Psychotherapy, University of Duisburg-Essen, Germany; 4 Department of Diagnostic and Interventional Radiology and Neuroradiology, University Hospital Essen, Germany; 5 Department of Psychiatry, Psychotherapy, and Psychosomatics, University Hospital Aachen, RWTH Aachen, Germany; 6 Department of Neuroradiology, University Hospital Innsbruck, Austria; 7 Division of Mind and Brain Research, Department of Psychiatry and Psychotherapy, Charité Universitätsmedizin Berlin, Berlin, Germany; George Mason University/Krasnow Institute for Advanced Study, United States of America

## Abstract

Men are traditionally thought to have more problems in understanding women compared to understanding other men, though evidence supporting this assumption remains sparse. Recently, it has been shown, however, that meńs problems in recognizing women’s emotions could be linked to difficulties in extracting the relevant information from the eye region, which remain one of the richest sources of social information for the attribution of mental states to others. To determine possible differences in the neural correlates underlying emotion recognition from female, as compared to male eyes, a modified version of the Reading the Mind in the Eyes Test in combination with functional magnetic resonance imaging (fMRI) was applied to a sample of 22 participants. We found that men actually had twice as many problems in recognizing emotions from female as compared to male eyes, and that these problems were particularly associated with a lack of activation in limbic regions of the brain (including the hippocampus and the rostral anterior cingulate cortex). Moreover, men revealed heightened activation of the right amygdala to male stimuli regardless of condition (sex vs. emotion recognition). Thus, our findings highlight the function of the amygdala in the affective component of theory of mind (ToM) and in empathy, and provide further evidence that men are substantially less able to infer mental states expressed by women, which may be accompanied by sex-specific differences in amygdala activity.

## Introduction

Sex differences in brain morphology and cognitive and affective functions have been increasingly documented [1]. One study has found that men have more problems in recognizing mental states and emotions in women than in men [2], and it is still unclear why this could be. Other studies have found that relative to women men are faster in detecting angry male faces [3]. Some authors have suggested that same-sex facial expressions may be more important to males as compared to females [4].

A so-called theory of mind (ToM) network and an empathy network of brain regions have been found to underlie the processes required to understand and share other people’s thoughts, feelings and intentions. The ToM network comprises of the following regions: the precuneus, posterior superior temporal sulcus (pSTS), temporo-parietal junction (TPJ), temporal poles, and medial prefrontal cortex (mPFC) [5], [6], whereas the empathy network includes the following brain areas: the anterior cingulate cortex (ACC) and the anterior insula [7]. However, the amygdala, which is also implicated in face processing [8], [9], also seems to play an important role in emotional empathy [10] and affective ToM [11].

Recent studies have suggested that deficits in recognizing facial emotions could be linked to difficulties in extracting the relevant information from the eye region (including gaze direction) [12], which remains one of the richest sources of social information for the attribution of mental states to others. Recent evidence has further indicated that social cognitive processes such as face processing and mental state attribution continue to develop during adolescence [13], and are due to genetic and/or hormonal (e.g. fetal testosterone) effects on brain maturation, which might develop distinctively in boys and girls [14], [15].

The Reading-the-Mind-in-the-Eyes Test (RMET) is based on photographs of isolated eye regions, and has been developed for the evaluation of mentalizing capacities in adults [16] and children [17]. In this test, four words indicating an emotional mental state are presented and subjects are required to choose which one best describes the emotion expressed in the eye region. A recent study using this test in a sample of university students has shown that men performed better in recognizing emotions from male vs. female eyes under placebo vs. vasopressin administration [2].

Based on these results, we expected that better performance in the recognition of emotions in male vs. female eyes would be related to differential (stronger) recruitment of networks underlying emotional empathy or affective ToM (i.e. the amygdala, anterior insula, and anterior cingulate cortex) [7], [10]. We examined a sample of healthy male participants using fMRI in combination with a modified version of the RMET [17], which required both sex discrimination and emotion recognition of the same pairs of female and male eyes.

## Materials and Methods

### Participants

22 single men aged between 21 and 52 years (mean age: 35.6±10.0) old were recruited for participation through advertisements. All participants were right-handed, medically and psychiatrically healthy, and with an estimated IQ [18] greater than 80 (mean IQ: 109.8±12.1). None of the subjects had any history of psychiatric illness, significant physical medical conditions, or any other condition that would interfere with MRI scanning (e.g., extreme obesity, claustrophobia, cochlear implant, metal fragments in eyes, cardiac pacemaker, neural stimulator, and metallic body inclusions or other metal implanted in the body). Following initial screening, subjects were interviewed about their medical history. Furthermore, the SCID I [19] and II [20] were administered by an experienced psychiatrist trained to use these instruments.

The study was approved by the local Committee on Medical Ethics of the Medical Faculty of the University of Duisburg-Essen, Germany and was performed in accordance with the Code of Ethics of the World Medical Association (Declaration of Helsinki). After a detailed description of the study was given to participants, written informed consent was obtained.

### fMRI Task

A modified version of the “Reading the Mind in the Eyes Test” (RMET)[16], [21]–[24], was administered to all participants in the scanner. The RMET assesses the ability to infer other people’s mental states, using information from 36 pairs of eyes (black-and-white pictures of eighteen male and eighteen female eyes). All stimuli were of equal size (22×8 cm) and depicted twelve negative, eight positive and sixteen neutral expressions, counterbalanced for the sex of the eyes [25].

Participants were asked to decide which of the two presented words (e.g., distrustful or terrified) best described the emotional/mental state of the person whose eyes were presented. The control condition comprised of a sex discrimination task in which for the same 36 pictures of eyes, the participants had to judge whether the eyes belonged to a man or woman. In total, the tasks consisted of 72 stimuli presented in a randomized (regarding stimuli within blocks and block order) block design with 12 blocks of six pairs of eyes. Participants responded via button press with their right index and middle fingers.

At the start of each block, the word “Emotion” or “Gender” was presented for seven seconds. The blocks assessing mental states were alternated with those assessing gender. Each picture was presented for eight seconds, and a question mark presented in the sixth second prompted the subjects to answer. Each block therefore lasted 48 seconds. Behavioral data were acquired with a button pad (Lumitouch™ Photon Control Inc, Burnaby, Canada). RMET scores were calculated as the percentage of correct discriminations over conditions, eye- type, and emotional valence as well as response times after the six-seconds period.

### Data Acquisition

All MR images were obtained using a 1.5 T MR (Sonata, Siemens, Erlangen, Germany) with a standard head coil. BOLD contrast images were acquired by applying an echo-planar acquisition technique (TR 3500 ms, TE 45 ms, flip angle 90°, FOV 240 mm, matrix 64) with 38 transversal slices (thickness 3.8×3.8×3 mm) and a 0.3 mm slice gap. Six initial “dummy” scans were eliminated prior to the data analysis to account for T1 relaxation effects.

### Image Processing

We used SPM8 software (http://www.fil.ion.ucl.ac.uk/spm/) for the analysis of the imaging data. Prior to second level statistical analyses, the images were realigned using sinc interpolation and normalized to the stereotactic template of the Montreal Neurological Institute (http://imaging.mrc-cbu.cam.ac.uk/imaging/MniTalairach). Bilinear interpolation was applied for normalization to the MNI-template. Normalized images were smoothed with an isotropic Gaussian kernel of 9 mm FWHM. Single subject contrasts between experimental and control conditions were computed. The model consisted of a boxcar function convolved with the hemodynamic response function (HRF) and the corresponding temporal derivative [26]. High-pass filtering with a cutoff frequency of 120 sec. and low-pass filtering with the HRF were applied.

### Statistical Analysis

#### Behavioral data

We performed a repeated measures analysis of variance (ANOVA) with two within subjects factors: (1) stimulus type (male eyes vs. female eyes) and (2) condition (emotion vs. gender recognition) in order to confirm that men had greater problems recognizing emotion than gender ( = main effect of condition) as well as to explore whether men reveal greater problems in recognizing emotions from female as compared to male eyes ( = condition x stimulus type interaction).

#### fMRI data

We used the framework of the General Linear Model to perform a statistical group analysis on the fMRI data on a voxel-by-voxel basis. Analyses included five mutually exclusive trial covariates: correct emotion recognition from male and female eyes as well as correct gender recognition from male and female eyes and errors (either from emotion or gender recognition trials). Partial correlation maps for individual participants were generated, indicating the extent to which each voxel**’**s activity conformed to an a priori canonical double gamma hemodynamic response function. Between-condition contrasts (i.e., gender vs. emotion recognition for all stimuli as well as male and female eyes separately) were estimated on a voxel-by-voxel basis in a whole brain approach. T Maps of activations for emotion and gender recognition in male and female eyes were submitted to random-effects ANOVAs to test for the main effect of condition (activation on emotion recognition minus activation on gender recognition) the main effect of stimulus type (male vs. female eyes), and the condition by stimulus type interaction among all subjects. Similar to earlier studies [22], [24], statistical thresholds were set to p<.001, uncorrected at the voxel level (height threshold) and p<.05, corrected after Family Wise Error (FWE) at the cluster level (extent threshold) in all whole-brain analyses. To increase sensitivity for stimulus and condition x stimulus interaction effects, we performed small volume corrections (SVC), if differential activations in these regions were predicted by our a priori hypothesis. For this purpose ROIs within areas related to affective empathy (i.e. the amygdala, anterior insula, and anterior cingulate cortex) were a priori defined by using the Automatic Anatomic Labeling (AAL) [27]. Spherical volumes with a 10-mm radius were centered on the coordinates of the peak voxel within the a priori defined ROI. Significance thresholds for both height and extent were set to p<.05 FWE corrected for multiple comparisons as well as the number of ROIs (3×2) at the voxel level.

Regression models were calculated to assess the associations of age with differences in performance accuracy and activation pattern. Activations in regions showing significant group differences were extracted by volume of interest analyses (1^st^eigenvariate) including all voxels of each cluster. Regression models were calculated step-wise with F probabilities of <.05 to enter and >.1 to remove variables. Multi co-linearity was tested by calculating the squared multiple correlation (R^2^) of predictor variables.

## Results

**Figure 1:**
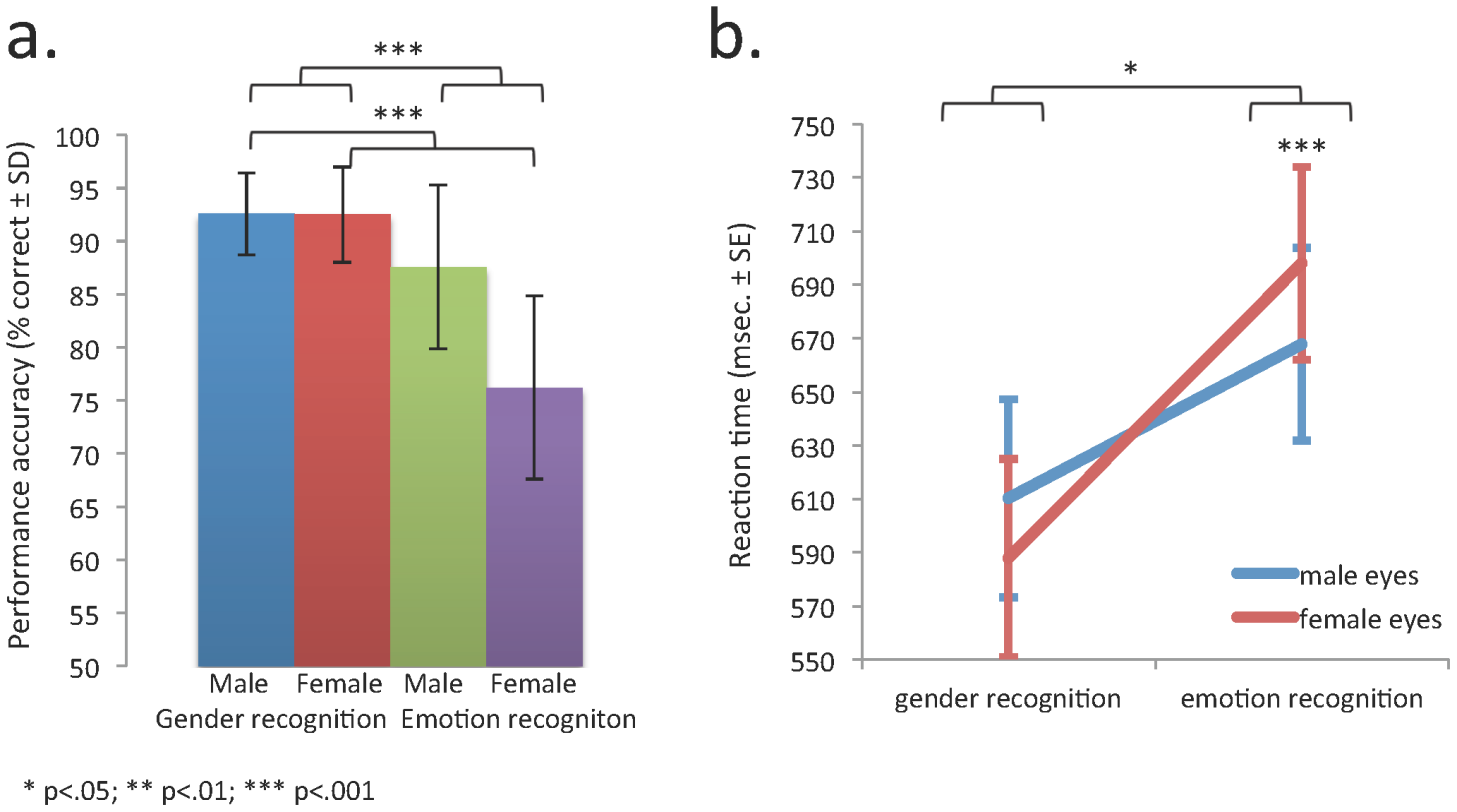
(a) Bar chart illustrating performance accuracy (% correct) and (b) line plot illustrating reaction time measures over conditions (emotion vs. gender recognition) and types of stimuli (male vs. female eyes). Significance bars and asterisks designate the significance of both recognition accuracy and reaction times for the main effects of condition and the condition by eyes type interactions.

As illustrated in Fig. 1a, men exhibited significantly greater problems recognizing emotion than gender (main effect of condition: F_1,21_ = 39.5; p<.001). Furthermore, they showed greater problems recognizing emotions from female compared to male eyes (condition x stimulus-type interaction: F_1,21_ = 24.1; p<.001). The same result was found for the reaction time measures (Fig. 1b). Men exhibited significantly longer reaction times when recognizing emotions than gender (main effect of condition: F_1,21_ = 16.0; p<.001) and the RT differences (emotion – gender recognition) were significantly larger for female than male eyes (condition x stimulus-type interaction: F_1,21_ = 4.5; p = .042). To confirm that differences in the ability to recognize emotions from male and female eyes were not affected by emotional valence of stimuli, an additional two (male vs. female eyes) by three (negative vs. positive vs. neutral expressions) ANOVA was performed. Neither a significant main effect for emotional valence (F_1,21_ = 0.54; p = .819) nor a significant eyes type by emotional valence interaction (F_1,21_ = 2.83; p = .107) could be detected.

**Figure 2:**
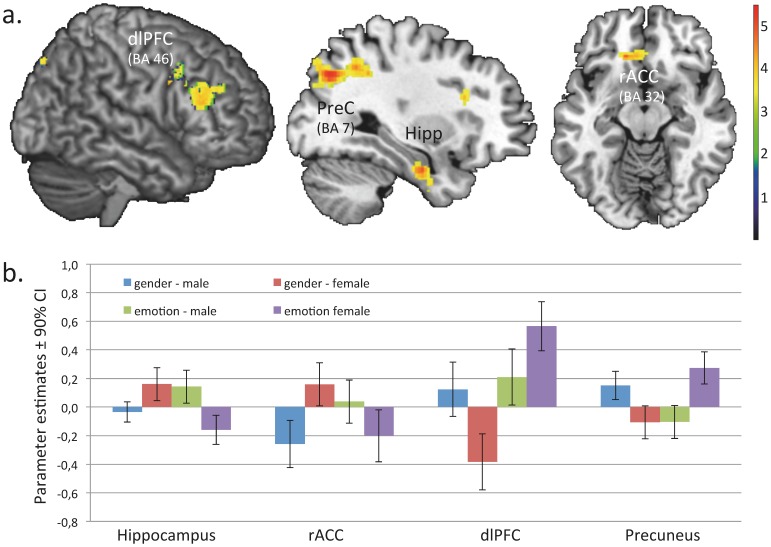
(a) Statistical parametric maps and (b) parameter estimates illustrating significant condition–by-stimulus-type interaction effects for the rACC (MNI: −6,30,−10; *p_FWE-SVC_* = 0.005; *z* = 3.85; cluster-size(*k*) = 194), right hippocampus (MNI: 32,−8,−26; p_FWEc_ = 0.025; *z* = 4.37; *k* = 130), precuneus (MNI: 28,−70,36; *p_FWEc_* = 0.034; *z* = 4.54; *k* = 494), and dlPFC (MNI: 52,32,22; *p_FWEc_* = 0.046; *z* = 3.85; *k* = 291). All coordinates reference the coordinate system of the Montreal Neurological Institute (MNI). Color bar indicates z*-*statistic value.

**Figure 3:**
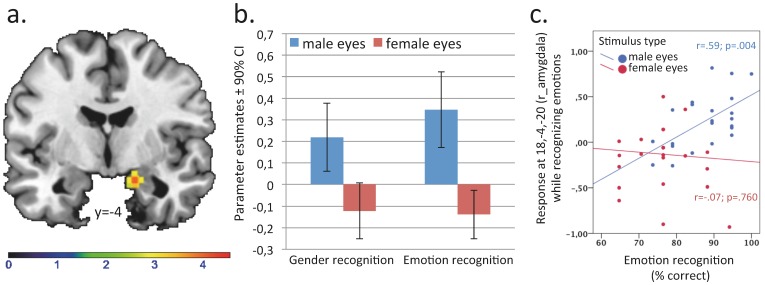
(a) Statistical parametric map and (b) parameter estimates illustrating differential activation pattern of the right amygdala (MNI: 18,−4,−20, *p_FWE-SVC_* = 0.004; *z* = 4.10; cluster-size = 69) for male and female eyes (regardless of condition: emotion vs. gender recognition). Color bar indicates z*-*statistic value. (c) Scatter plots depicting the relationships between amygdala response and emotion recognition accuracy for both types of stimuli.

Emotion as compared to gender recognition led to significant activation of the left inferior frontal gyrus (pars orbitalis and triangularis; extending into the anterior insula and temporal pole; MNI: −50, 28,−8; p_FWEc_<.001; *z* = 6.03; *k* = 3182), the bilateral pSTS/TPJ (MNI: −58, −42,6; p_FWEc_<.001; *z* = 5.78; *k* = 1407; MNI: 52, −36,−2; p_FWEc_ = 0.032; *z* = 4.70; *k* = 200), and the mPFC/ACC (MNI: −2, 20,54; p_FWEc_ = 0.048; *z* = 4.36; *k* = 160). There were also activations in the bilateral occipital cortex (MNI: −16, −98,−8; p_FWEc_ = 0.037; *z* = 4.06; *k* = 192; MNI: 32, −90,−12; p_FWEc_ = 0.173; *z* = 3.87; *k* = 118), of which the right cluster passed the significance threshold at the voxel but not at the cluster level. As illustrated in Fig. 2a, there were significant condition-by-stimulus-type interactions for activation of the right precuneus, right dorsolateral PFC (dlPFC), right hippocampus, and left rostral ACC (rACC; details regarding coordinates and statistics are provided in the figure legend). Contrast estimates as provided in Fig. 2b showed that interaction effects of the hippocampus and rACC reflect increased activation while recognizing emotions from male as compared to female eyes, whereas interaction effects of the precuneus and the dlPFC reflect the opposite pattern. Interestingly, there was also a significant main effect for stimulus type in the right amygdala (MNI: 18,−4,−20, *p_FWE-SVC_* = 0.004; *z* = 4.10; cluster-size = 69), reflecting increased activation to male relative to female eyes regardless of condition (see Fig. 3).

Since right amygdala activation to male eyes was higher compared to female eyes regardless of condition, but also seemed to be descriptively higher during the emotion recognition condition, we tested whether right amygdala activation modulated recognition accuracy. We therefore extracted non-adjusted activation (main effect: male vs. female stimuli) of the cluster within the right amygdala using volume of interest analysis (1^st^ eigenvariate) and calculated Pearsońs correlation coefficient between right amygdala responses and recognition accuracy for male and female stimuli separately. There was a significant positive association between right amygdala activation and mentalizing performance for male (r = .59; p = .004), but not female stimuli (r = -.07; p = .760; see Fig. 3c).

Given that previous research has documented a negative association between age and mentalizing performance [28], we explored whether stimulus-related differences in mentalizing performance and neural responses were linked to age. There was a negative association between age and mentalizing performance (beta = -.466; p = .029) as well as differential right amygdala activity (beta = -.419; p = .049). Yet, performing additional analyses of covariance including age as a covariate, neither the condition-by-stimulus-type interaction on recognition accuracy (F_1,20_ = 5.0; p = .009) nor differential right amygdala activity (MNI: 18,−2,−20, *z* = 3.9) were solely a consequence of its association with age.

### Post-hoc Analysis

Since previous research has shown a relationship between amygdala activity and pupil diameter changes in faces with different emotions [29], [30], we analyzed whether pupil diameter size differed due to eye type, emotional valence or both. A two (eye type) by three (emotional valence) ANOVA on pupil diameter sizes, however, revealed neither a significant main effect of eye type (F_6,30_ = 2.29; p = .118), or emotional valence (F_6,30_ = 1.84; p = .184) nor a significant eye type by emotional valence interaction (F_6,30_ = 0.71; p = .500). Thus, it seems unlikely that the finding of different responsiveness of the right amygdala to male vs. female eyes is the result of differences in pupil diameter size.

## Discussion

In this study, we confirmed recent research documenting that men actually have greater difficulties to accurately assess emotional expressions via female as compared to male eyes [2]. In addition, we showed for the first time that better performance in recognizing emotions from male vs. female eyes was linked to differential activation patterns in areas that have been previously linked to mentalizing performance [2], [3]. While the main effect of condition confirmed previous research documenting that emotion recognition, relative to gender, recruited areas of the mentalizing network (including pSTS/TPJ, temporal poles, and mPFC/ACC) as well as areas predicting the affective consequences of a stimulus (i.e. the anterior insula [7]), the condition-by-stimulus-type interaction revealed significant effects in four regions (i.e. hippocampus, rostral ACC, dlPFC, and precuneus), indicating differential activation while recognizing emotions as compared to gender in both types of stimuli (male vs. female). Emotion vs. gender recognition in male relative to female eyes was associated with heightened activation in the right hippocampus and the rostral ACC, whereas emotion vs. gender recognition in female relative to male eyes was associated with heightened activation of the right dlPFC and the right precuneus. Moreover, we found that right amygdala responses were increased for male stimuli regardless of condition (emotion vs. gender recognition).

Both the hippocampus and the rACC are involved in the acquisition and expression of emotional memories [31]. Thus, increased activation of these regions during recognition of emotions vs. gender in male relative to female eyes may reflect relatively stronger use of autobiographical memory information when taking others meńs perspective. Furthermore, Perry et al. reported increased hippocampus activation during an emotional mentalizing task and suggested that in order to better understand and empathize with others, memories are projected to people that are seen as similar to oneself [32]. Though they used a different task, a similar phenomenon might have occurred in our subjects, thus revealing higher hippocampus activation during recognition of male eyes, possibly due to a higher attribution of similarity as compared to females. Furthermore, activation in rACC has been related to self-referential processing [33], [34]. Emotion recognition might depend on self-referential processing since, in order to be successful, the observer tries to covertly mimic the other person’s mental state, which then results in shared mental states between observer and observed person [34]. Together with pSTS and anterior insula activation during emotion vs. gender recognition (regardless of stimulus type), this network might aid in inferring mental states, and thus predicting other people’s beliefs and intentions by drawing inferences from one’s own and other’s past and present social information [35].

By contrast, from the regions that showed heightened activation while inferring mental states vs. gender from female as compared to male eyes, the precuneus has been repeatedly shown in studies on theory of mind but also in studies on the self, whereas the dlPFC has been reported during self-regulation [33], [34], [36]. Furthermore, the dlPFC and the precuneus have shown strong interconnections [24], and have been related to episodic memory [36], [37]. Therefore, their enhanced co-activation during emotion recognition of female eyes in our male subjects might indicate an increased attempt for memory retrieval from previous encounters with females in which the recognition of the specific emotion had occurred.

The finding of heightened right amygdala responses during recognition of male compared to female stimuli might indicate a highly automated and stimulus-driven effect that occurred regardless of different conditions or instructions. Thus, increased right amygdala responses to male stimuli may indicate a sex-specific association between stimulus type (male vs. female) and automated emotion processing or affective empathy. The positive correlation between right amygdala responses and the ability to infer mental states from male but not female eyes also indicated that affective empathy might enhance mentalizing performance for male stimuli in men. Conversely, this indicated that the processing of opposite-sex stimuli might be associated with lower affective empathy which may also be associated with a reduced mentalizing ability. In support of this assumption, a previous study investigating emotion recognition accuracy in two patients with acquired amygdala lesions showed impaired emotion recognition using an earlier version of the RMET [38]. Results from this study particularly showed impairment in the male patient with right amygdala damage, whereas the female patient with bilateral amygdala lesion did not present any mentalizing impairments. Notably, both patients made comparable numbers of errors on items on the RMET [28].

Moreover, it is possible that the pattern of seemingly decreased right amygdala (from the main effect of male vs. female eyes) and increased right dlPFC activation while inferring emotions from female relative to male eyes, reflected a down-regulation mechanism of emotional experiences specific to female stimuli, which bears an additional source of information for the attribution of mental states to women. If true, one would expect a negative correlation between activation of the right dlPFC and the amygdala, which is exactly what we found. We observed a significant negative correlation of -.438 (p = .042) between activation of the right dlPFC and right amygdala during emotion recognition. Thus, this mechanism might account for the relatively greater problems men have in recognizing women’s compared to men’s emotions.

Interestingly, intranasal administration of nonapeptide arginine-vasopressin (AVP) has been shown to negatively affect meńs emotion recognition accuracy to same- but not opposite-sex stimuli [2]. AVP is an evolutionarily highly conserved mediator in the regulation of complex social cognition and behavior. Most brain imaging studies support the view that the effects of oxytocin and, with less evidence, AVP on social processing and particularly the emotional component of empathy are mediated by limbic circuitry, with the amygdala as a core structure [39]. Thus differences in emotion recognition accuracy to same- vs. opposite-sex eyes might be associated with vasopressin modulated changes in the amygdala.

Mentalizing allows people to empathize with others in order to predict their beliefs, intentions or behavior, or even to deceive others when needed [33]. Thus, our findings highlight the functional significance of the amygdala, particularly for the affective components of ToM and empathy, and reveal further evidence that men are less able to infer mental states expressed by women, which may be accompanied by an amygdala-related deficit to affectively empathize with women. The finding that men are superior in recognizing emotions/mental states of other men, as compared to women, might be surprising. From an evolutionary point of view, accurate interpretations of other men’s rather than women’s thoughts and intentions, especially threatening cues (also related to amygdala responsiveness [40]), may have been a factor contributing to survival in ancient times. As men were more involved in hunting and territory fights, it would have been important for them to be able to predict and foresee the intentions and actions of their male rivals.

In further support of this notion, it has been found that, when compared to women, men are better at identifying facial expressions of anger [41], while women are more superior in recognizing fear and sadness [41], [42]. Consequently, exposure to angry male as opposed to angry female faces has been shown to lead to enhanced physiological arousal [43] as well as activation of the ACC [44] in men but not in women.

Since the neural basis of the mentalizing network has also been found to be active in studies on self-referential processing, as well as episodic memory, it is most likely that the ‘core function of this network is to draw inferences on self- as well as other-related social information in the past, present and future. This simulation enables sharing the other’s state based upon one’s own previous experience and knowledge’ [35]. In evolutionary terms, it also makes sense that these mentalizing processes operate best in situations when the other is most similar to oneself.
